# Uptake of radiolabeled GlcNAc into *Saccharomyces cerevisiae* via native hexose transporters and its *in vivo* incorporation into GPI precursors in cells expressing heterologous GlcNAc kinase

**DOI:** 10.1111/j.1567-1364.2011.00778.x

**Published:** 2012-01-18

**Authors:** John J Scarcelli, Paul A Colussi, Anne-Lise Fabre, Eckhard Boles, Peter Orlean, Christopher H Taron

**Affiliations:** 1 New England BiolabsIpswich, MA, USA; 2 Institut für Molekulare Biowissenschaften, Johann Wolfgang Goethe-Universität FrankfurtFrankfurt, Germany; 3 Department of Microbiology, University of Illinois at Urbana-ChampaignUrbana, IL, USA

**Keywords:** glycosylphosphatidylinositol, chitin, phosphoethanolamine, mannosyltransferase, biosynthesis, hexose transporter

## Abstract

Yeast glycan biosynthetic pathways are commonly studied through metabolic incorporation of an exogenous radiolabeled compound into a target glycan. In *Saccharomyces cerevisiae* glycosylphosphatidylinositol (GPI) biosynthesis, [^3^H]inositol has been widely used to identify intermediates that accumulate in conditional GPI synthesis mutants. However, this approach also labels non-GPI lipid species that overwhelm detection of early GPI intermediates during chromatography. In this study, we show that despite lacking the ability to metabolize *N*-acetylglucosamine (GlcNAc), *S. cerevisiae* is capable of importing low levels of extracellular GlcNAc via almost all members of the hexose transporter family. Furthermore, expression of a heterologous GlcNAc kinase gene permits efficient incorporation of exogenous [^14^C]GlcNAc into nascent GPI structures *in vivo*, dramatically lowering the background signal from non-GPI lipids. Utilizing this new method with several conditional GPI biosynthesis mutants, we observed and characterized novel accumulating lipids that were not previously visible using [^3^H]inositol labeling. Chemical and enzymatic treatments of these lipids indicated that each is a GPI intermediate likely having one to three mannoses and lacking ethanolamine phosphate (Etn-P) side-branches. Our data support a model of yeast GPI synthesis that bifurcates after the addition of the first mannose and that includes a novel branch that produces GPI species lacking Etn-P side-branches.

## Introduction

Glycosylphosphatidylinositol (GPI) is a complex glycolipid attached to the C-terminus of certain eukaryotic secretory pathway proteins that anchors them to the exterior face of the plasma membrane. GPI anchor precursors are synthesized in the endoplasmic reticulum (ER) by successive addition of components to phosphatidylinositol (PI), after which completed GPI precursors are transferred *en bloc* to proteins by the GPI transamidase complex. GPI anchored-proteins (GPI-APs) then proceed to the plasma membrane via the secretory pathway (for reviews see Orlean & Menon, 2007; Paulick & Bertozzi, 2008). The process of forming GPI precursors is essential, as deletion of genes for enzymes responsible for the early stages of GPI anchor biosynthesis results in lethality in yeasts (Leidich *et al*., 1994; Colussi & Orlean, 1997; Grimme *et al*., 2004) and mammals (Kawagoe *et al*., 1996).

The core structure of the GPI anchor is protein-Etn-P-6Manα1-2Manα1-6Manα1-4GlcN-inositol-PO_4_-lipid. A fourth mannose is present as an α1,2-linked side-branch of the third mannose (Man-3) on *Saccharomyces cerevisiae* GPIs but may be added in a tissue-specific manner in mammals (Taron *et al*., 2004). Additionally, in yeast and mammals, the innermost two mannoses of GPIs may be modified by side-branching phosphoethanolamine (Etn-P) residues. While addition of Etn-P is required for proper yeast GPI precursor synthesis, the order of addition of Etn-P to the first mannose (Man-1) and of the second mannose (Man-2) to GPI precursors is not yet clear. The essential Mcd4 protein has been implicated in the addition of Etn-P to Man-1 of yeast GPIs (Gaynor *et al*., 1999; Flury *et al*., 2000). Both a conditional *mcd4* mutant (Wiedman *et al*., 2007) and cells treated with the putative Mcd4p inhibitor YW3548 (Sütterlin *et al*., 1997, 1998) accumulate a GPI intermediate having two mannoses and lacking a side-branching Etn-P (termed an ‘un-substituted’ Man_2_-GPI), suggesting that Etn-P addition to Man-1 occurs after Man-2 addition. However, when yeast cells are depleted of the second GPI mannosyltransferase (Gpi18p), a GPI intermediate lipid that bears a single mannose with a side-branching Etn-P accumulates (Fabre *et al*., 2005), suggesting that Mcd4p is capable of adding Etn-P prior to Man-2 addition. One possible explanation for these contradictory phenotypes is that Mcd4p and Gpi18p may both be capable of acting on a common substrate, a GPI intermediate with a single un-substituted mannose (Man_1_-GPI). However, direct observation of Man_1_-GPI in either *mcd4* or *gpi18* cells has not previously been possible because of limitations of the analytical methods used for yeast GPI intermediate characterization.

Elucidation of yeast GPI synthesis has involved the characterization of lipids that accumulate in strains having conditional defects in various steps of the pathway (Orlean & Menon, 2007). Analysis of GPI pathway intermediates is challenging because they do not accumulate in amounts sufficient to permit their direct analysis using mass spectrometry. Thus, studies reporting characterization of yeast GPI pathway intermediates have relied upon *in vivo* incorporation of a radiolabeled component into GPI structures to permit their visualization by thin layer chromatography (Sipos *et al*., 1994; Hamburger *et al*., 1995; Canivenc-Gansel *et al*., 1998; Sütterlin *et al*., 1998; Benachour *et al*., 1999; Taron *et al*., 2000, 2004; Grimme *et al*., 2001; Fabre *et al*., 2005; Wiedman *et al*., 2007). [^3^H]Inositol is most commonly used to metabolically radiolabel yeast GPIs because it is both efficiently taken up by cells and incorporated into GPI structures. However, this method also produces an abundance of [^3^H]phosphatidylinositol and [^3^H]inositol-containing phosphoceramides that co-migrate with GPI intermediates on thin layer chromatograms and obscure detection of less polar species such as Man_1_-GPI. In principle, to visualize these intermediates, other compounds such as [^3^H]mannose or [^3^H]glucosamine (GlcN) could be employed. Indeed, incorporation of [^3^H]mannose into GPIs has been accomplished in yeast but requires the use of strains carrying a *pmi40* mutation that blocks entry of [^3^H]mannose into central metabolism (Payton *et al*., 1991; Sipos *et al*., 1994; Taron *et al*., 2000). However, our prior attempts to introduce *pmi40* into *S. cerevisiae mcd4* and *gpi18* mutant backgrounds resulted in synthetic lethality (A. Fabre and C. Taron, unpublished observations). Furthermore, uptake of [^3^H]mannose or [^3^H]GlcN is poor, even when glucose concentration in the medium is kept low. Finally, the use of radiolabeled GlcNAc to visualize GPI intermediates has not been successful because *S. cerevisiae* lacks a GlcNAc salvage pathway that would facilitate its conversion to UDP-GlcNAc.

In this study, we systematically examined the requirements for import and incorporation of GlcNAc into GPI structures in *S. cerevisiae*. We demonstrate that despite lacking a GlcNAc-specific plasma membrane transporter, *S. cerevisiae* cells are capable of importing significant quantities of GlcNAc via endogenous hexose transporters. In light of this finding, we show that expression of a single heterologous gene encoding GlcNAc kinase is the only requirement to permit cells to efficiently incorporate radiolabeled GlcNAc from the medium into GPI anchors. Importantly, few non-GPI lipids become radiolabeled, thus permitting improved visualization of early GPI intermediates. Using this novel approach, we examined the lipid accumulation phenotypes of several known GPI biosynthesis mutants that are conditionally defective in assembly of the GPI glycan. Our data permit us to resolve unanswered questions regarding the timing and order of Man-2 and Etn-P addition to Man-1 of yeast GPIs. Our data support a revised yeast GPI synthesis model that bifurcates immediately after the addition of the first mannose and that may include a pathway branch that produces GPI precursors entirely lacking Etn-P side-branches on Man-1 and Man-2.

## Materials and methods

### Materials

[2-^3^H] *myo*-inositol (sp. act. 15–20 Ci mmol^−1^), [1,6-^3^H] *N*-acetyl-d-glucosamine (sp. act. 30–60 Ci mmol^−1^), and [1-^14^C] *N*-acetyl-d-glucosamine (sp. act. 50–60 mCi mmol^−1^) were obtained from American Radiolabeled Chemicals (St. Louis, MO). Jack bean alpha mannosidase (JBαM) and phosphatidylinositol-specific phospholipase C (PI-PLC) were obtained from Sigma-Aldrich (St. Louis, MO), and *Aspergillus saitoi* α1,2 mannosidase mannosidase was from ProZyme (Hayward, CA). *Xanthomonas manihotis* α1,2-3 mannosidase, α1,6 mannosidase, and all restriction endonucleases were from New England Biolabs (Ipswich, MA).

### Yeast strains, media, and culturing conditions

*Saccharomyces cerevisiae* strains BY4743, VW1000, *mcd4-174* (GY1450), *gpi18Δ*-pGAL-*GPI18*, *smp3-2*, *gpi10Δ*-pGAL-*GPI10* have been described previously (Brachmann *et al*., 1998; Sütterlin *et al*., 1998; Gaynor *et al*., 1999; Wieczorke *et al*., 1999; Grimme *et al*., 2001; Fabre *et al*., 2005). Yeast strains were routinely propagated in synthetic medium (Difco™ yeast nitrogen base medium; Beckton, Dickinson & Co., Sparks, MD) containing the nutritional supplements needed to complement strain auxotrophies, or rich medium (1% yeast extract and 2% peptone). Media were supplemented with 2% carbon source (glucose, GlcNAc, or galactose) and 2% agar in the case of solid media.

To compare the growth of individual strains on agar plates, similar amounts of cells of each strain were picked from agar plates and suspended in 0.25 mL liquid synthetic medium lacking a carbon source and supplements (NCS medium) to a final concentration of 10^8^ cells mL^−1^. One hundred microliters of each suspension was placed in a microtiter plate well, and 10-fold serial dilutions were made in fresh NCS medium. Aliquots of each dilution (5 mL) were spotted onto synthetic agar medium plates containing appropriate nutritional supplements and 2% carbon source and then incubated at 30 °C for 2–4 days.

To compare the growth rates of individual strains, cells were cultured in 40 mL synthetic medium containing either 2% glucose (SGlc medium) or 2% GlcNAc (SGlcNAc medium) and required nutritional supplements in 250 mL baffled flasks. Cultures were grown at 30 °C for 120 h with shaking at 280 r.p.m. Cell density was determined by measuring light absorbance at 600 nm in an Ultraspec 2100 Pro spectrophotometer (GE Healthcare, Piscataway, NJ).

### Expression of *Candida albicans* NAG genes in *S. cerevisiae*

The *C. albicans* genes *CaNAG5* (GenBank XM_707193), *CaNAG2* (XM_707196), *CaNAG1* (XM_707195), and *CaNGT1* (XM_712674) were each expressed in *S. cerevisae* BY4734 from the endogenous *PGK1* promoter (Pro_*PGK1*_). Expression cassettes for each gene were assembled using a multi-step ‘PCR knitting’ strategy. In the first step, each gene was amplified from *C. albicans* CAI4 genomic DNA in a manner that introduced the terminal ∼50 bp of the *S. cerevisiae PGK1* promoter to the 5′ end of each gene. Additionally, *S. cerevisiae* Pro_*PGK1*_ was amplified from vector pGBN1_PGK_ (Colussi & Taron, 2005) in four separate reactions that each introduced DNA corresponding to the first ∼50 bp of *CaNAG5*, *CaNAG2*, *CaNAG1*, or *CaNGT1* to the 3′ end of the promoter. Finally, each amplified *C. albicans* gene was fused to its corresponding Pro_*PGK1*_ fragment by ‘PCR knitting’. In this reaction, overlapping regions of homologous DNA present on amplified genes and promoter fragments anneal and become extended to create a small amount of full-length template fragment, which is subsequently amplified for cloning. Amplification of gene and promoter fragments was performed using Deep Vent™, Taq or Phusion™ DNA polymerases (New England Biolabs) as recommended by the supplier. ‘PCR knitting’ of gene and promoter fragments was performed using Phusion™ DNA polymerase with ∼200 ng each of promoter and gene amplicons present as template.

Amplified Pro_*PGK1*_-*CaNAG5*, Pro_*PGK1*_-*CaNAG2*, Pro_*PGK1*_-*CaNAG1*, and Pro_*PGK1*_-*CaNGT1* expression cassettes were digested with NotI and BamHI and cloned into the NotI-BamHI sites of the yeast shuttle vectors pRS415 (CEN, *LEU2*), pRS413 (CEN, *HIS3*), pRS416 (CEN, *URA3*), and pRS414 (CEN, *TRP1*), respectively (Sikorski & Hieter, 1989). All primers used in assembly of these constructs are described in the Supporting information (Appendix S1).

### Expression of *S. cerevisiae* hexose transporter genes

Individual hexose transporter genes were expressed from the *S. cerevisiae MET25* promoter as previously described (Wieczorke *et al*., 1999; Hamacher *et al*., 2002). Each vector was individually introduced into strain VW1000, and import of [^3^H]GlcNAc by each resulting strain was measured as described later.

### [^3^H]GlcNAc cellular import assay

Strains were grown to a cell density of approximately 5 × 10^6^ cells mL^−1^ in SGlc medium and required nutritional supplements. Harvested cells were washed twice with 10 mL of NCS medium and re-suspended in NCS medium to a density of 1 × 10^8^ cells mL^−1^. Five microliters (5 μCi) of [^3^H]GlcNAc was added to triplicate wells of a 96-well plate followed by the addition of 100 μL of cells to each well (∼1 × 10^7^ cells per well). The mixtures were incubated for 1 h at RT before the contents of each well were applied to GF/C glass micro-filters (Whatman, Florham Park, NJ) under vacuum to immobilize cells. Each filter was washed five times with 1 mL NCS medium before being transferred to vials containing 1.5 mL of scintillation fluid. Radioactivity was measured for each sample using a Tri-Carb 2900TR scintillation counter (PerkinElmer, Waltham, MA), and the total pmol of sugar internalized by cells was determined by comparison with a standard curve generated with [^3^H]GlcNAc.

### Metabolic labeling of lipids with [^14^C]GlcNAc and [^3^H]inositol

To prepare cells for [^14^C]GlcNAc labeling, they were first grown to saturation in synthetic medium containing 2% glucose (for *smp3-3* cells) or 2% galactose (for *mcd4-174*, *gpi18Δ*-pGAL-*GPI18*, and *gpi10Δ*-pGAL-*GPI10* cells), 0.2% Difco™ yeast extract (Becton, Dickinson & Co.), and the supplements needed to complement strain auxotrophies. Culturing was performed at 25 °C for *smp3-2* cells and at 30 °C for the *mcd4-174*, *gpi18Δ*-pGAL-*GPI18* and *gpi10Δ*-pGAL-*GPI10* strains. Cells were then diluted to an OD_600 nm_ of 0.1 and grown to OD_600 nm_ 0.6–1.0, after which they were harvested by centrifugation and washed twice with fresh synthetic medium containing 2% of the appropriate carbon source (glucose or galactose) but lacking yeast extract.

Temperature-sensitive *smp3-2* and *mcd4-174* cells were resuspended in 1 mL of fresh SGlc medium then shifted to nonpermissive temperature (37 °C) for 20 min prior to the addition of 2 μCi [^14^C]GlcNAc and incubation for 2 h with shaking. Labeling of these strains under permissive conditions was performed the same manner but at 25 °C for *smp3-2* cells and at 30 °C for *mcd4-172* cells. Strains that permit conditional depletion of Gpi18p or Gpi10p in the presence of glucose (*gpi18Δ*-pGAL-*GPI18* and *gpi10Δ*-pGAL-*GPI10*) were resuspended in 1 mL of fresh SGlc medium for 20 min prior to the addition of 2 μCi [^14^C]GlcNAc and incubation for 2 h at 30 °C with shaking. Labeling of these strains under permissive conditions was performed in medium containing 2% galactose (SGal) in place of glucose.

After labeling, cells were harvested by centrifugation and lipids were extracted as described later. Metabolic labeling of lipids with 5 μCi [^3^H]inositol was performed in inositol-free synthetic medium as previously described (Taron *et al*., 2000).

### Measurement of cellular chitin

Quantitation of [^14^C]GlcNAc incorporation into cell wall chitin was performed using an adaptation of an alkali extraction method (Bulik *et al*., 2003). Specifically, 25 OD_600 nm_ units of cells were metabolically labeled with [^14^C]GlcNAc as described earlier and collected by centrifugation at 15 800 ***g*** for 2 min. The cell pellet was washed three times with deionized water (dH_2_O) to remove unincorporated label, then was re-suspended in 1 mL 6% KOH, and incubated at 80 °C for 90 min with occasional mixing. Insoluble chitin was collected by centrifugation at 15 800 ***g*** for 5 min. The pellet was washed three times with 0.5 mL dH_2_O, re-suspended in 0.2 mL 8 M HCl, and incubated for 4 h at 102 °C to hydrolyze the chitin and release soluble [^14^C]GlcNAc. A 100 μL aliquot of this hydrolysate was added to 1.5 mL scintillation fluid, and radioactivity was measured with a Tri-Carb 2900TR scintillation counter (PerkinElmer).

### Isolation and separation of GPI lipid intermediates

GPI lipid intermediates were extracted from metabolically labeled cells in chloroform/methanol/water (10 : 10 : 3, v/v/v) and partitioned in 1-butanol as previously described (Taron *et al*., 2000). GPI lipid intermediates were analyzed by separation on silica 60 thin layer chromatography (TLC) plates (EM Science, Darmstadt, Germany). TLC plates were prerun in solvent A (chloroform/methanol/water, 65 : 25 : 4 v/v/v) after which lipids were applied and resolved in either solvent B (chloroform/methanol/water, 5 : 5 : 1, v/v/v), solvent C (chloroform/methylene/chloride/methanol/water, 5 : 5 : 10 : 2, v/v/v/v), solvent D (chloroform/methanol/water, 10 : 10 : 2.5 v/v/v), or solvent E (chloroform/methanol/water, 10 : 10 : 3 v/v/v). TLC-separated radiolabeled lipids were visualized by fluorography using a BioMax Transcreen LE intensifier screen (Eastman Kodak, Rochester, NY) and BioMax MS film (Kodak).

### Chemical and enzymatic treatment of GPI lipid intermediates

Lipids isolated from approximately 20 OD_600 nm_ units of metabolically radiolabeled *S. cerevisiae* cells were treated with methanolic ammonia (mild base) or PI-PLC as previously described (Taron *et al*., 2000). Treatment of lipids with JBαM was performed by resuspending vacuum-dried lipids in 100 μL of 0.1 mM zinc acetate (pH 7.5) containing 3 M ammonium sulfate, 1% (v/v) Nonidet P-40 Substitute (Sigma), and 0.5 U JBαM. Reactions were incubated at 37 °C overnight. Treatment of lipids with 20 U *X. manihotis* α1-2,3 mannosidase (Xmα2M) or 8 U *X. manihotis* α1-6 mannosidase (Xmα6M) was performed in 100 mM sodium phosphate (pH 5) containing 5 mM CaCl_2_, 0.1% (w/v) sodium deoxycholate for ∼17 h at 37 °C, after which the reactions were spiked with the same amount of buffer and fresh enzyme and allowed to continue for an additional 24 h at 37 °C.

Following enzyme or chemical treatments, reactions were dried by vacuum evaporation and lipids were desalted by partitioning in 1-butanol as previously described (Taron *et al*., 2000) prior to analysis by TLC.

## Results

### Assimilation of GlcNAc by *S. cerevisiae*

**Fig 1:**
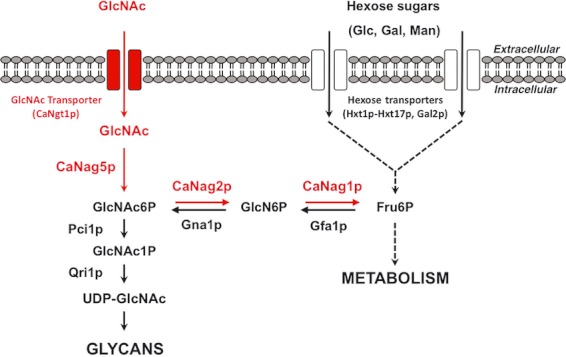
Yeast GlcNAc metabolism. *Candida albicans* imports and metabolizes GlcNAc via a salvage pathway (red text and arrows) that is absent from *Saccharomyces cerevisiae*. This pathway consists of a GlcNAc-specific transporter (CaNgt1p), GlcNAc kinase (CaNag5p), GlcNAc6P deactylase (CaNag2p), and GlcN6P deaminase (CaNag1p). *Saccharomyces cerevisiae* cannot metabolize GlcNAc, and instead essential UDP-GlcNAc is formed *de novo* from Fru6P (black text and arrows), an intermediate in the metabolism of various hexose sugars (e.g., Glc, glucose; Gal, galactose; Man, mannose). Hexoses are internalized by *S. cerevisiae* cells via several plasma membrane hexose transporters (the Hxt family and Gal2p).

*Saccharomyces cerevisiae* and *C. albicans* differ in their ability to assimilate various hexoses; *S. cerevisiae* is unable to utilize GlcNAc as a sole carbon source, whereas *C. albicans* can. This is because of the absence of a GlcNAc salvage pathway in *S. cerevisiae* (Fig. 1). Recent studies have used expression of *C. albicans* GlcNAc salvage pathway genes in *S. cerevisae* to generate strains capable of growth on GlcNAc as a sole carbon source (Wendland *et al*., 2009) or capable of incorporating synthetic GlcNAc analogs into glycoproteins (Breidenbach *et al*., 2010). Prior studies have reported that *S. cerevisiae* lacks the ability to import significant amounts of GlcNAc (Singh & Datta, 1979; Alvarez & Konopka, 2007). Additionally, *S. cerevisiae* lacks obvious homologs of *C. albicans* GlcNAc-specific membrane transporters (e.g., CaNgt1p). Thus, in both GlcNAc salvage pathway engineering studies, co-expression of a *C. albicans* GlcNAc-specific transporter was used to ensure the import of GlcNAc into cells prior to its conversion to UDP-GlcNAc or entry into central metabolism. One drawback to this approach was that elevated expression of *CaNGT1* in *S. cerevisiae* was toxic (Breidenbach *et al*., 2010) and therefore could be problematic if *CaNGT1* were expressed in conditional *gpi* mutants, many of which already exhibit significant growth defects. Thus, we investigated whether expression of GlcNAc salvage pathway enzymes in the absence of a GlcNAc-specific transporter could still permit growth of *S. cerevisiae* on GlcNAc.

**Fig 2:**
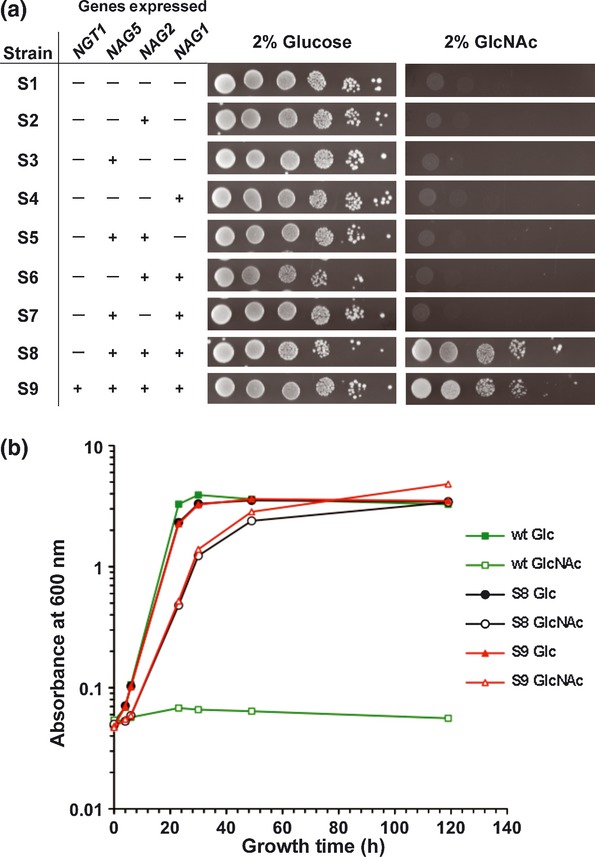
Growth of engineered *Saccharomyces cerevisiae* strains on GlcNAc. (a) *Saccharomyces cerevisiae* strains S1–S9 expressing different combinations of *Candida albicans* GlcNAc salvage pathway genes (NGT1, NAG5, NAG2, and NAG1) were propagated on synthetic agar medium containing 2% glucose or 2% GlcNAc. Patches represent 10-fold serial dilutions of cells prepared as described in the Materials and methods section. (b) The culture density of wt (S1), S8 and S9 strains in liquid synthetic medium containing 2% glucose or 2% GlcNAc measured over 100 h of shaking at 30 °C.

*Candida albicans* salvage pathway genes (*CaNAG5*, *CaNAG2*, *CaNAG1*) were individually cloned downstream of the *PGK1* promoter in different centromeric vectors (see Materials and methods). The resulting expression plasmids were introduced in all combinations into wild-type *S. cerevisiae* BY4734 cells. In strains where an expression vector was omitted, the corresponding empty vector was introduced in its place. The resulting strains were tested for their ability to grow on synthetic medium containing either 2% glucose or 2% GlcNAc as a sole carbon source (Fig. 2a). Notably, a strain co-expressing all three genes (strain S8) was able to grow on medium containing GlcNAc, despite the absence of a heterologous GlcNAc transporter (*CaNGT1*). Additional co-expression of CaNgt1p in strain S8 (forming strain S9) did not further improve the strain's growth rate on GlcNAc medium despite increasing the rate at which these cells internalized [^3^H]GlcNAc from 0.016 to 0.445 pmol GlcNAc per minute (data not shown), indicating that GlcNAc import is not a limiting factor for GlcNAc assimilation in this background. Growth curve analysis indicated that strains S8 and S9 both grew slightly slower on GlcNAc (∼110 min doubling time) than on glucose (∼75 min doubling time) (Fig. 2b).

These data support the conclusion that *S. cerevisiae* cells naturally possess a mechanism for importing small quantities of GlcNAc. Furthermore, the amount of GlcNAc internalized by *S. cerevisiae* is sufficient to sustain vegetative growth when a functional salvage pathway is present.

### GlcNAc import into *S. cerevisiae* cells

**Fig 3:**
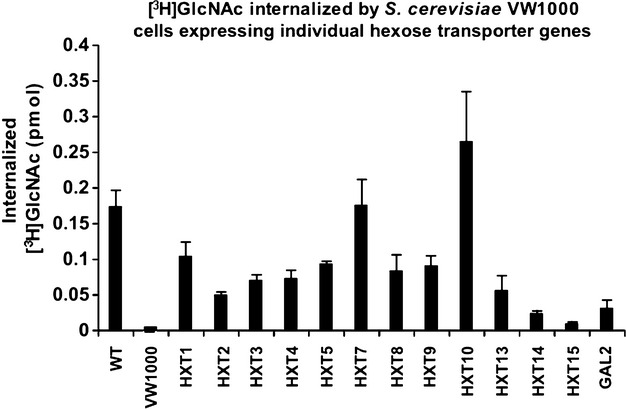
Internalization of [^3^H]GlcNAc by *Saccharomyces cerevisiae* cells. Import of extracellular [^3^H]GlcNAc into the cytoplasm was compared between wild-type cells, cells genetically deleted of all hexose transporter genes (strain VW1000), and VW1000 cells over-expressing individual hexose transporter genes (HXT genes and GAL2). HXT6, HXT11, HXT17, and HXT16 were not included in the study as they are nearly identical to HXT7, HXT9, HXT13, and HXT15, respectively. HXT12 is a pseudogene.

To understand better how *S. cerevisiae* imports GlcNAc, we examined the uptake of [^3^H]GlcNAc in strains defective in the membrane transport of hexose sugars. *Saccharomyces cerevisiae* is capable of importing hexoses to varying degrees by way of membrane transporters encoded by the *HXT* gene family (see Leandro *et al*., 2009 for a review). Additionally, galactose is internalized by the membrane transporter Gal2p (Reifenberger *et al*., 1997; Leandro *et al*., 2009). VW1000 is an *S. cerevisiae* strain in which all *HXT* genes and *GAL2* have been genetically deleted (Wieczorke *et al*., 1999). Although this strain is unable to utilize glucose and galactose for growth, it can be propagated on medium containing maltose. In this strain background, cellular import of [^3^H]GlcNAc was entirely abolished (Fig. 3, lane 2) indicating that GlcNAc uptake is entirely dependent upon one or more hexose transporters. To further define which specific hexose transporters were capable of GlcNAc import, various VW1000 strains each harboring a vector directing over-expression of a single transporter gene from the strong *MET25* promoter (Wieczorke *et al*., 1999; Hamacher *et al*., 2002) were tested for their ability to internalize [^3^H]GlcNAc (Fig. 3, lanes 3–15). In this experiment, Gal2p and all hexose transporters except Hxt14p and Hxt15p showed an ability to import [^3^H]GlcNAc into the cytoplasm, with Hxt7p and Hxt10p being the most efficient.

### Incorporation of exogenous GlcNAc into chitin in engineered *S. cerevisiae*

*Saccharomyces cerevisiae* incorporates GlcNAc into asparagine-linked glycans (*N*-glycans), GPI anchors, and the cell wall polysaccharide chitin. In all cases, UDP-GlcNAc is the GlcNAc donor for the various glycosyltransferases that synthesize these glycans. In wild-type *S. cerevisiae*, synthesis of UDP-GlcNAc occurs through sequential modification of the glycolytic intermediate Fru6P (black text in Fig. 1). However, because our data indicated that GlcNAc can be internalized by endogenous hexose transporters, we reasoned that expression of heterologous GlcNAc kinase (*CaNAG5*) alone may permit direct conversion of exogenous GlcNAc to UDP-GlcNAc via GlcNAc6P.

**Fig 4:**
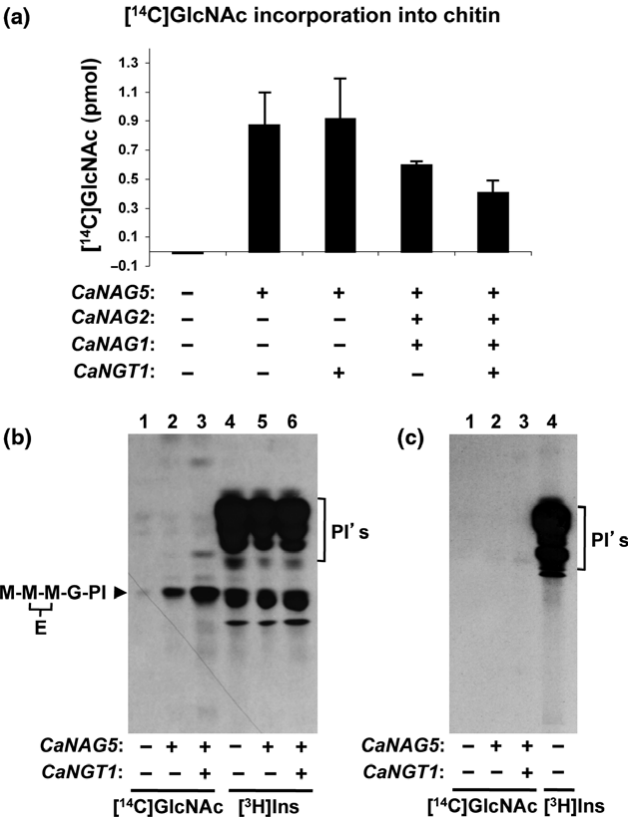
Incorporation of [^14^C]GlcNAc into *Saccharomyces cerevisiae* polysaccharides. (a) *Saccharomyces cerevisiae* strains expressing different combinations of *Candida albicans* GlcNAc salvage pathway genes were exposed to extracellular [^14^C]GlcNAc. Cell wall chitin was isolated from each strain, and the amount of [^14^C]GlcNAc incorporated into chitin was measured by scintillation counting. (b) A fluorograph showing the TLC separation of [^14^C]GlcNAc or [^3^H]inositol-labeled lipids isolated from smp3 cells or (c) wild-type cells expressing various combinations of CaNAG5 and CaNGT1. Solvent B was used for separation (see Materials and methods). The position of a characterized Man_3_(Etn-P)-GPI lipid that accumulates in smp3 cells is indicated (Grimme *et al*., 2001). M, mannose; E, ethanolamine phosphate; G, glucosamine; PI, phosphatidylinositol, Ins, inositol.

Chitin is a long polymer of unbranched β1,4-linked GlcNAc residues and is a critical structural component of the yeast cell wall. The process of synthesizing chitin through the action of chitin synthases is the largest consumer of the cellular UDP-GlcNAc pool in yeast (Milewski *et al*., 2006). Therefore, we measured the incorporation of exogenous [^14^C]GlcNAc from the growth medium into cell wall chitin as a means of quantifying the efficiency at which internalized [^14^C]GlcNAc becomes converted to UDP-[^14^C]GlcNAc. No [^14^C]GlcNAc became incorporated into chitin extracted from wild-type cells (Fig. 4a, lane 1), whereas significant quantities of label were incorporated into chitin in all strains that expressed *CaNAG5* (Fig. 4a, lanes 2–5). Additionally, co-expression of the CaNgt1p GlcNAc-specific transporter did not significantly increase the incorporation of [^14^C]GlcNAc into chitin (Fig. 4a, lane 3). Finally, in strains capable of GlcNAc metabolism because of co-expression of CaNag5p, CaNag2p, and CaNag1p (Fig. 4a, lanes 4 and 5), a decrease in labeled chitin was observed. This is consistent with the possibility that in these strains, internalized [^14^C]GlcNAc can either be incorporated into chitin via UDP-GlcNAc or enter central metabolism via Fru6P. Considered together, these data clearly demonstrate that the minimum requirement for the efficient incorporation of [^14^C]GlcNAc into chitin via UDP-GlcNAc is expression of *CaNAG5*. Further, these data also suggest that other *S. cerevisiae* glycans that receive GlcNAc via UDP-GlcNAc (e.g., *N*-glycans, GPI anchors) will also become radiolabeled *in vivo* in cells expressing *CaNAG5* and fed [^14^C]GlcNAc.

### Detection of GPI synthesis intermediates with [^14^C]GlcNAc

Because GlcNAc is associated with few non-GPI lipids, we reasoned that [^14^C]GlcNAc labeling might give rise to less background and improve visualization of less polar GPI species compared with [^3^H]inositol labeling. To test this notion, a haploid strain carrying a temperature-sensitive (*ts*) allele of *smp3*, the gene encoding the yeast GPI fourth mannose transferase, was subjected to metabolic labeling with [^14^C]GlcNAc both with and without expression of *CaNAG5*. The *smp3* mutant is known to prominently accumulate Man_3_(Etn-P)-GPI (Grimme *et al*., 2001) that is readily observed with [^3^H]inositol labeling (Fig. 4b, lanes 4–6). This lipid was also observed after [^14^C]GlcNAc labeling when *CaNAG5* was expressed (Fig. 4b, lanes 2–3) and only a trace was seen in lipids from cells not expressing *CaNAG5* (Fig. 4b, lane 1). Furthermore, in a control experiment, no [^14^C]GlcNAc-containing lipids accumulated in wild-type cells expressing *CaNAG5* and *CaNGT1* (Fig. 4c, lanes 1–3).

In *smp3* cells labeled with [^14^C]GlcNAc, co-expression of the *CaNGT1* GlcNAc transporter gene appeared to enhance the amount of Man_3_(Etn-P)-GPI observed via [^14^C]GlcNAc labeling (Fig. 4b, lane 3 vs. lane 2). However, in subsequent experiments with other *gpi* mutants, expression of *CaNGT1* in addition to *CaNAG5* did not further improve the incorporation of [^14^C]GlcNAc into GPI intermediates (data not shown). Thus, in all subsequent experiments, only *CaNAG5* expression was used for [^14^C]GlcNAc labeling of GPI intermediates. Finally, compared with [^3^H]inositol labeling, far fewer [^14^C]GlcNAc labeled, non-GPI lipids were observed, resulting in much clearer visualization of accumulating GPI species. Thus, we conclude that [^14^C]GlcNAc can be diverted into GPI pathway intermediates upon *CaNAG5* expression in yeast *gpi* mutants, and that this labeling method can be superior to [^3^H]inositol labeling for the visualization of GPI intermediate lipids in yeast because it allows less polar GPIs to be detected. Furthermore, [^14^C]GlcNAc labeling of GPIs is effective when carried out in medium containing 2% glucose, whereas the visualization of glycolipids by [^3^H]mannose labeling in the *pmi40* background is typically performed in medium containing 0.1% glucose (Sipos *et al*., 1994; Taron *et al*., 2000).

### Visualization of mannosylated GPIs lacking Etn-P using [^14^C]GlcNAc labeling

**Fig 5:**
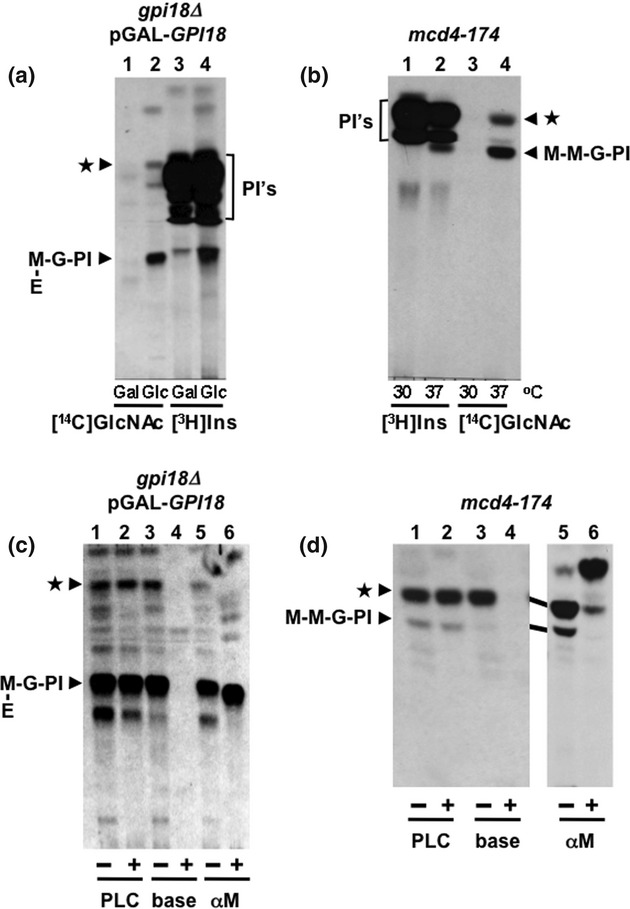
Accumulation of an un-substituted Man_1_-GPI in gpi18 and mcd4 mutants. (a,b) Conditional gpi18 and mcd4 mutants expressing CaNAG5 were metabolically labeled with [^14^C]GlcNAc or [^3^H]inositol ([^3^H]Ins) under permissive (a, Gal or b, 30 °C) and nonpermissive (a, Glc or b, 37 °C) conditions. Extracted lipids were TLC separated and detected by fluorography. (c,d) [^14^C]GlcNAc-labeled lipids isolated from gpi18 and mcd4 cells were subjected to chemical and enzymatic treatments prior to TLC separation. In all panels, the positions of known Man_1_(Etn-P)-GPI and Man_2_-GPI species that accumulate in gpi18 and mcd4 are shown. These analyses confirm the presence of a Man_1_-GPI (black star) accumulating in both gpi18 and mcd4 cells. TLC separation for a, b, c, and d was performed in solvents B, C, B, and D, respectively (see Isolation and separation of GPI lipid intermediates). PLC, phospholipase C; base, mild base hydrolysis; αM, jack bean α-mannosidase; M, mannose; E, ethanolamine phosphate; G, glucosamine; PI, phosphatidylinositol.

Because of the dramatic reduction in the *in vivo* labeling of non-GPI lipids with [^14^C]GlcNAc compared with [^3^H]inositol, [^14^C]GlcNAc was utilized to better characterize the lipid accumulation phenotypes of early-stage GPI biosynthesis mutants. In these experiments, *CaNAG5* was expressed in *mcd4* and *gpi18* conditional mutants and each strain was subjected to metabolic labeling with [^14^C]GlcNAc. For each mutant, previously characterized Man_1_(EtnP)-GPI and Man_2_-GPI intermediates (that accumulate in *gpi18* and *mcd4* cells, respectively) were prominently observed (Fig. 5a and b). Additionally, each mutant also showed the accumulation of a second less polar lipid that migrated similarly to [^3^H]inositol-labeled PI species (Fig. 5a and b, black star). These two new [^14^C]GlcNAc-labeled lipids had identical *R*_f_ values and co-migrated on TLC (data not shown). Each lipid was both resistant to phospholipase C (PI-PLC) and sensitive to treatment with mild base (Fig. 5c and d, black star), a combination of characteristics observed only with GPI synthesis intermediates. Furthermore, each was sensitive to JBαM indicating that its head group contained a terminal mannose lacking a side-branching Etn-P (Fig. 5c, lane 6; Fig. 5d, lane 6). Finally, each lipid migrated as a more hydrophobic species than the Man_2_-GPI lacking Etn-P side-branches that accumulates in the *mcd4-174* mutant (Wiedman *et al*., 2007). Considered together, these data support the conclusion that the unknown lipid observed in both mutants is an early GPI intermediate with a single mannose lacking an Etn-P side-branch.

**Fig 6:**
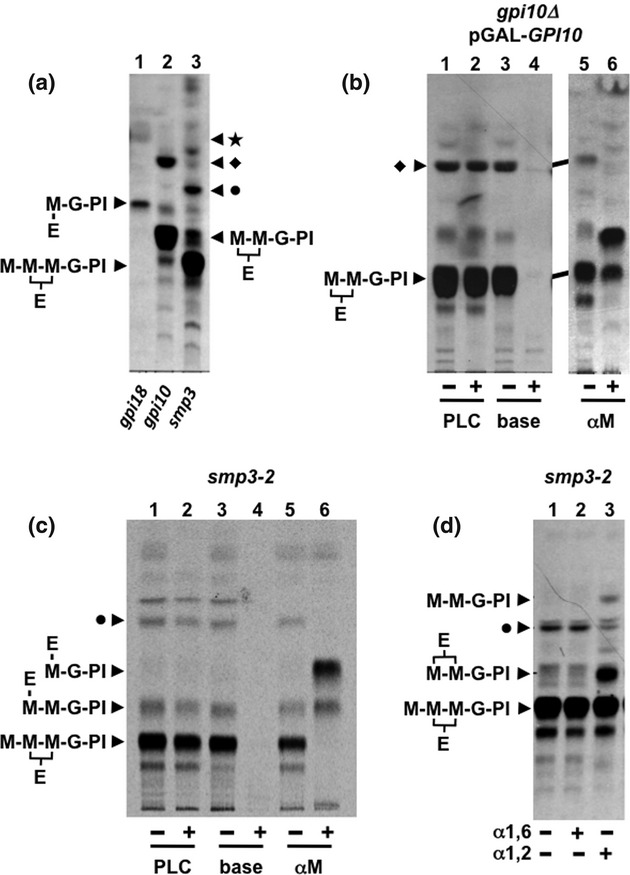
Accumulation of un-substituted GPI intermediates in later-stage GPI synthesis mutants. (a) TLC separation (in solvent E) of [^14^C]GlcNAc-labeled GPI intermediates that accumulate in gpi18, gpi10, and smp3 mutants under nonpermissive conditions. The positions of putative un-substituted Man_1_-, Man_2_-, and Man_3_-GPIs are denoted with a black star, diamond and circle, respectively. The structures and positions of known GPI intermediates are also shown. (b,c) TLC separation (in solvent B) of [^14^C]GlcNAc-labeled lipids from gpi10 and smp3 mutants after chemical or enzymatic treatment. (d) Characterization of a putative un-substituted Man_3_-GPI (black circle) that accumulates in smp3 cells by digestion with α1,6 and α1,2 mannosidases. TLC separation was performed with solvent B. PLC, phospholipase C; base, mild base hydrolysis; αM, jack bean α-mannosidase, M, mannose; E, ethanolamine phosphate; G, glucosamine; PI, phosphatidylinositol.

To determine whether yeast cells can extend the Man_1_-GPI glycan *in vivo* without side-branching Etn-P addition, we performed [^14^C]GlcNAc labeling analyses with two later-stage GPI synthesis mutants, *gpi10* and *smp3*. These mutants are conditionally defective in the addition of Man-3 and Man-4 to GPI precursors and accumulate Man_2_(Etn-P)-GPI and Man_3_(Etn-P)-GPI, respectively (Canivenc-Gansel *et al*., 1998; Sütterlin *et al*., 1998; Grimme *et al*., 2001). [^14^C]GlcNAc labeling revealed that each mutant also shows a low abundance accumulation of a second lipid (Fig. 6a, back diamond and circle). These lipids were each sensitive to mild base, resistant to PI-PLC digestion and sensitive to JBαM digestion (Fig. 6b and c) indicating they are each GPI intermediates lacking Etn-P moieties. Furthermore, the unknown lipid from *gpi10* cells co-migrates on TLC with the previously characterized un-substituted Man_2_-GPI that accumulates in *mcd4* cells (data not shown). Considered together, the likeliest interpretation of these observations is that the un-substituted GPI intermediate that accumulates in *gpi10* cells is a Man_2_-GPI.

The accumulation of Man_1_-GPI and Man_2_-GPI in *gpi18* and *gpi10* cells, respectively, suggested that the lipid accumulating in *smp3* cells might be an un-substituted Man_3_-GPI. To test this notion, we sought evidence that this lipid's glycan head group was terminated with an un-substituted α1,2-linked mannose (the linkage of Man-3) by mannosidase digestion. We found the commonly used *A. saitoi* α1,2 mannosidase to be completely inactive in buffer containing the detergent needed to disperse the lipid. However, *X. manihotis* α1,2-3 (XmαM2) mannosidase was partially active in detergent but required long incubations to achieve partial mannose removal. Extended incubation of [^14^C]GlcNAc-labeled *smp3* lipids with XmαM2 revealed significant reduction in the un-substituted lipid (Fig. 6d, lane 3) and appearance of an un-substituted Man_2_-GPI product, consistent with the partial removal of a terminal α1,2-linked mannose. Thus, we conclude that this lipid is likely to be a Man_3_-GPI lacking Etn-P side-branches.

## Discussion

In this study, we developed a method to direct efficient incorporation of radiolabeled GlcNAc into GlcNAc- or GlcN-containing glycans *in vivo* in *S. cerevisiae*. This was made possible by our new findings that *S. cerevisiae* cells have an innate ability to import small quantities of extracellular GlcNAc via endogenous plasma membrane hexose transporters and that expression of a heterologous GlcNAc kinase gene is the only requirement for the incorporation of extracellular GlcNAc into chitin and GPI pathway intermediates.

We utilized this method to identify novel accumulating lipids in several known *S. cerevisiae gpi* assembly mutants that were not previously detectable by [^3^H]inositol labeling. We showed that these lipids were structurally consistent with each being a GPI synthesis intermediate having from one to three mannoses and lacking Etn-P side-branches on Man-1 or Man-2. Furthermore, the formation of these lipids suggests that *S. cerevisiae* GPI synthesis may include a pathway branch that produces un-substituted GPI precursors, although the physiological significance of such lipids is currently unclear.

### Early steps in yeast GPI synthesis

The order of addition of the second GPI mannose (by Gpi18p) and an Etn-P residue side-branched to the first mannose (by Mcd4p) in *S. cerevisiae* GPI assembly is still poorly defined. Several lines of evidence have suggested that yeast Etn-P transferase Mcd4p prefers a Man_2_-GPI as substrate: conditionally lethal *mcd4* cells accumulate a Man_2_-GPI *in vivo* (Wiedman *et al*., 2007), a Man_2_-GPI is the largest intermediate formed in a cell-free GPI synthesis assay using *mcd4* membranes (Zhu *et al*., 2006), and yeast cells treated with the putative Mcd4p/PIG-N inhibitor YW3548 accumulate a Man_2_-GPI (Sütterlin *et al*., 1997, 1998). However, we previously reported that cells conditionally depleted of the second GPI mannosyltransferase (Gpi18p) accumulate a Man_1_(Etn-P)-GPI suggesting that Mcd4p is also capable of adding Etn-P to a GPI intermediate having only one mannose (Fabre *et al*., 2005).

The possibility that Gpi18p and Mcd4p can both act on a Man_1_-GPI has been discussed (Orlean & Menon, 2007), although such a Man_1_-GPI had not been previously observed in yeast. Our *in vivo* [^14^C]GlcNAc labeling experiments with *mcd4* and *gpi18* has revealed that both mutants indeed accumulate a Man_1_-GPI lacking Etn-P on its mannose.

### Formation of yeast late-stage GPI intermediates lacking Etn-P

The accumulation of Man_2_-GPI in *mcd4* and *gpi10* cells (Wiedman *et al*., 2007) and the inability of cell-free GPI synthesis to proceed past Man_2_-GPI in *mcd4* membranes (Zhu *et al*., 2006) have suggested that the addition of Etn-P to Man-1 is strictly required for the addition of the third mannose by Gpi10p. However, this requirement is not absolute because the lethality of *mcd4* deletion can be bypassed *in vivo* by over-production of Gpi10p (Wiedman *et al*., 2007). One interpretation of this observation is that Gpi10p may have a preference for a Man_2_(Etn-P)-GPI substrate but can also weakly transfer mannose to the un-substituted Man_2_-GPI that is produced in the absence of Mcd4p. In support of this notion, we have shown direct evidence of an un-substituted Man_3_-GPI via [^14^C]GlcNAc labeling of cells defective in the addition of a fourth mannose (*smp3-2*) to GPI precursors. This lipid is significantly less abundant than the Man_3_(Etn-P)-GPI species that predominantly accumulates in *smp3-2* cells. Additionally, accumulation of an un-substituted Man_4_-GPI has been reported in Gpi13p-depleted cells (Benachour *et al*., 1999). The formation of unsubstituted Man_1_-, Man_2_-, Man_3_-, and Man_4_-GPI intermediates *in vivo* is consistent with the possibility that Gpi18, Gpi10, and Smp3 mannosyltransferases can all use GPI intermediates lacking Etn-P as acceptors; however, it cannot be ruled out that these unmodified GPIs could also arise from aberrant removal of Etn-P from GPIs having side-branched Etn-P residues.

### Yeast GPI synthesis model

**Fig 7:**
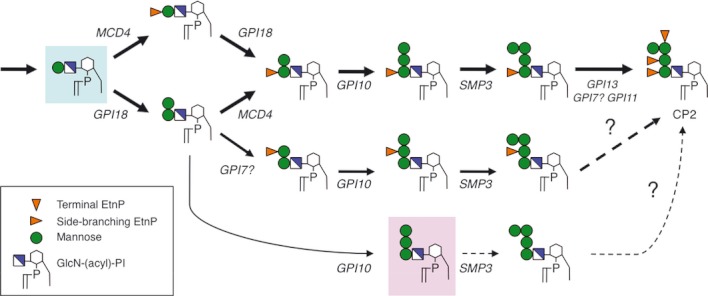
A revised model for yeast GPI glycan synthesis. A branched GPI glycan synthesis pathway is suggested from the structures of lipids that accumulate in various conditional yeast GPI synthesis mutants. Two new lipids lacking Etn-P were identified in this study, a Man_1_-GPI (blue highlight) and a Man_3_-GPI (pink highlight). Formation of these lipids suggests that Mcd4p and Gpi18p do not act in a specific order and provides a route to the putative synthesis of Man_4_-GPIs that lack Etn-P side-branches. It is currently unclear whether structures lacking Etn-P on Man-1 are ultimately converted to the complete precursor CP2. Arrow thickness reflects relative differences in lipid abundance observed in each pathway branch.

The steps of yeast GPI glycan synthesis have been characterized largely through the structural determination of GPI intermediates that accumulate in mutants conditionally defective in different pathway steps. Understanding the timing and order of the steps of GPI glycan assembly and Etn-P side-branch addition has been complicated by the observation that these mutants typically accumulate several isoforms of a GPI lipid that differ in the number or position of Etn-P side-branches on Man-1 and Man-2. For example, yeast cells defective in the addition of the essential fourth mannose to GPIs (*smp3*) accumulate three distinct isoforms of a trimannosyl GPI intermediate, one with a single Etn-P side-branched to Man-1 (Grimme *et al*., 2001), one with a single Etn-P side-branched to Man-2 (Grimme *et al*., 2001), and one lacking any Etn-P residues (described in this study). Multiple accumulating lipids have also been observed in *gpi10*, *gpi11*, and *gpi13* conditional mutants (Benachour *et al*., 1999; Taron *et al*., 2000; Wiedman *et al*., 2007). The pathway model shown in Fig. 7 seeks to reconcile the various structures previously observed in GPI glycan synthesis mutants with the observations of this study.

The current thinking is that the complete precursor that becomes attached to proteins in yeast has four mannoses with side-branching Etn-P residues on both Man-1 and Man-2, and a ‘terminal’ Etn-P residue on Man-3, through which the precursor becomes linked to protein (Fig. 7). Several observations from studies with both yeast and mammalian cells support this notion. In mammalian cells, the presence of Etn-P on Man-1 is likely a prerequisite for the association of GPI precursors with the GPI transamidase complex that transfers them to proteins (Vainauskas & Menon, 2006). Additionally, removal of Etn-P from Man-2 of mammalian protein-bound GPIs permits exit of the GPI anchored protein from the ER (Fujita *et al*., 2009). Finally, Mcd4p-mediated addition of Etn-P to Man-1 of yeast GPIs is required for remodeling of GPI lipids to ceramides and for efficient transport of GPI-APs from the ER to the Golgi (Zhu *et al*., 2006). Thus, it appears that Etn-P substitution of Man-1 and Man-2 are likely important attributes of most protein-bound GPIs. However, we cannot exclude the possibility that lower abundance isoforms of the complete precursor bearing one or no Etn-P side-branches could be transferred to proteins. Alternatively, these lipids could stay protein-free and serve some cellular function distinct from that of protein-bound GPIs.

In summary, we have shown that most of *S. cerevisiae*'s hexose transporters can mediate GlcNAc uptake, and we have developed a novel approach for efficiently channeling radiolabeled GlcNAc into yeast GPI intermediates via UDP-GlcNAc. Using this method to examine the lipid accumulation profiles of known conditional GPI biosynthesis mutants, we have discovered novel GPI intermediate lipids that lack Etn-P side-branches on their innermost two mannoses. The identification of these lipids has helped clarify early events in GPI glycan synthesis and provides evidence that the yeast GPI assembly pathway includes a branch that produces un-substituted GPI precursors.

## Statement

Re-use of this article is permitted in accordance with the Terms and Conditions set out at http://wileyonlinelibrary.com/onlineopen#OnlineOpen_Terms

## Supporting Information

Appendix S1. Primers used in assembly of NAG gene expression vectors. Please note: Wiley-Blackwell is not responsible for the content or functionality of any supporting materials supplied by the authors. Any queries (other than missing material) should be directed to the corresponding author for the article.
