# Supplementary material for: Uptake of radiolabeled GlcNAc into Saccharomyces cerevisiae via native hexose transporters and its in vivo incorporation into GPI precursors in cells expressing heterologous GlcNAc kinase
Source: FEMS Yeast Res. 2012 Jan 18;12(3):305–16. doi: 10.1111/j.1567-1364.2011.00778.x (PMC3498731; doi:10.1111/j.1567-1364.2011.00778.x)
Supplement: Appendix S1. — Primers used in assembly of NAG gene expression vectors. Please note: Wiley-Blackwell is not responsible for the content or functionality of any supporting materials supplied by the authors. Any queries (other than missing material) should be directed to the corresponding author for the article. [file fyr0012-0305-sd1.pdf]

Primers used in "PCR knitting" assembly of DNA constructs having each NAG gene cloned downstream of the S. cerevisiae PGK1 promoter– In a first round of PCR, four separate reactions were used to amplify the *S. cerevisiae PGK1* promoter while introducing 3' tails homologous to the 5' ends of *CaNAG5*, *CaNAG2*, *CaNAG1* or *CaNGT1*. Each reaction contained the forward primer PGK-F: 5'-ATAAGAAT<u>GCGGCCGC</u>GTGGCCTCTTATCGAGAAAGAAAT-3' and one of the following reverse primers:

PGK-R1 (CaNAG5)
5'-ACGCAACCCACTAATGCTAGTCTCAGTCATTGTTTTATATTTGTTGTAAAAAGTAGATAA-3'

PGK-R2 (CaNAG2)
5'-ATGACAGTTTGTGAATCTAGTAAATGACATTGTTTTATATTTGTTGTAAAAAGTAGATAA-3'

PGK-R3 (CaNAG1)
5'-GTTAGGGTTGGAAAATATAGCTTGTCTCATTGTTTTATATTTGTTGTAAAAAGTAGATAA-3'

PGK-R4 (CaNGT1)
5'-AATATCCATTTTAGTTTGATCTTTCTCCATTGTTTTATATTTGTTGTAAAAAGTAGATAA-3'

In a second round of PCR, four separate reactions were used to amplify *CaNAG5*, *CaNAG2*, *CaNAG1* and *CaNGT1* in manner that introduced a tail homologous to the 3' end of the *S. cerevisiae PGK1* promoter upstream of each gene's start codon.

CaNAG5 was amplified with the forward primer NAG5-F:
5'-TTATCTACTTTTTACAACAAATATAAAACAATGACTGAGACTACATTAGTGGGTTGCGT-3'
and the reverse primer NAG5-R:
5'-CGC<u>GGATCC</u>CTACTTATGATAGGCAGCACCTATGGC-3'

CaNAG2 was amplified with the forward primer NAG2-F:
5'-TTATCTACTTTTTACAACAAATATAAAACAATGTCATTTACTAGATTCACAAACTGTCAT-3'
and the reverse primer NAG2-R:
5'-CGC<u>GGATCC</u>CTATAAAACAGCAGTTAATTTATC-3'

CaNAG1 was amplified with the forward primer NAG1-F:
5'-TTATCTACTTTTTACAACAAATATAAAACAATGAGACAAGCTATATTTTCCAACCCTAAC-3'
and the reverse primer NAG1-R:
5'-CGC<u>GGATCC</u>CTACAACTTTGACTTTAATCCAGCGGC-3'

CaNGT1 was amplified with the forward primer NGT1-F:
5'-TTATCTACTTTTTACAACAAATATAAAACAATGGAGAAAGATCAAACTAAAATGGATATT-3'
and the reverse primer NGT1-R:
5'-CGC<u>GGATCC</u>TTACTCAATATGTACTGTTGTTGA-3'

The positions of engineered NotI and BamHI sites used for cloning into yeast shuttle vectors are an underline.
